# Knowledge, Attitudes, and Practices of Primary School Teachers Regarding Deleterious Oral Habits Leading to Malocclusion in School Children: A Cross-Sectional Study

**DOI:** 10.7759/cureus.75130

**Published:** 2024-12-05

**Authors:** Kowmudi Maddineni, Parvez Sulthana, Swapna Sannapureddy, Kiranmayi Govula, Prasanth Punamalli, Balajiyaswanth Sajja

**Affiliations:** 1 Conservative Dentistry and Endodontics, Narayana Dental College and Hospital, Nellore, IND; 2 Dentistry, Narayana Dental College and Hospital, Nellore, IND; 3 Public Health Dentistry, Narayana Dental College and Hospital, Nellore, IND

**Keywords:** lip biting, malocclusion, nail biting, oral habits, thumb-sucking, tongue thrusting

## Abstract

Background

Oral habits are most initiated at the primary school age. Primary school children are seen to have been performing these habits during their active school hours. The peak days they are in school are the most active hours during which the tendency to perform this habit occurs. So, most primary school teachers encounter students in their classes with such habits. Hence, primary school teachers play a vital role in developing healthy habits.

Objective

The study aims to assess the knowledge, attitude, and awareness of primary school teachers regarding the oral habits of children that lead to malocclusion.

Study setting and design

The study was a cross-sectional descriptive observational study of primary school teachers of Nellore, Andhra Pradesh. The study used a questionnaire survey, and different domains were studied, such as the primary school teachers' knowledge, attitudes, and practices. The pretested questionnaires, which involved paper and pen, were distributed to the study participants in person. The filled questionnaires were collected on the same day. A total of 150 responses were collected, with a response rate of 100%. The filled data were gone through to sort out the data obtained as the percentage through every question. The data thus obtained in terms of percentage were analyzed statistically.

Results

Among 150 teachers, 104 (69.3%) observed the harmful effects of oral habits in children, with 99 (66%) noticing the abnormal alignment of teeth in children's dentition. One hundred ten (73.3%) knew the disbenefits caused by pernicious oral habits.

Conclusions

Within the limitations, most school teachers in Nellore had less knowledge about oral habits. Their attitude toward preventing oral habits was appreciable. The practice domain showed that many teachers had tried to prevent children from continuing their oral habits. Many teachers do not have enough knowledge to educate or explain the need to consult or visit a dentist, indicating that there is a need to implement training and awareness programs for them. All these findings from this study convey a message to public health dentistry-related professionals about their roles.

## Introduction

Oral habits lead to teeth malalignment, which plays a vital role in malocclusion. Oral habits such as thumb-sucking and digit sucking, tongue thrusting, lip sucking, cheek biting, nail biting or pencil biting, and mouth breathing are pernicious habits among children of the age group 5-12 years [1,2]. The children of this age group have these oral habits, which affect tooth positioning because of the disturbed eruption forces induced on the soft and hard tissue of the oral cavity by these habits [3]. Thumb-sucking can potentially result in digital hyperextension, which may necessitate surgical correction. Thumb-sucking can also cause paronychia, which is a type of infection. According to Birra et al., most infants will begin to suck their fingers and thumbs voluntarily in their early months of life, and many have been observed to do so in utero [4]. Alopecia, which develops in thumb-sucking children who concurrently pull on, twist, and pull off their hair, is another physical risk related to thumb-sucking. The detrimental effects of these oral habits are the anterior open bite of various grades resulting in the protruded maxillary arch or collapsed arch form in the anterior region or posterior region of multiple degrees, which, if established, will lead to inefficient chewing of the food.

Oral habits in children develop due to various insecurities psychologically, and if these are left unattended, they become habituated, leading to malocclusion. These insecurities develop in children usually during their preschool age due to the nonavailability of the protective environment that was so far available when at home [5]. School teachers should know these oral habits and their harmful effects on developing dentition. As children of this age spend most of their active time at school, the teachers play a crucial role in desisting these pernicious oral habits [6]. Hence, this study was designed to assess school teachers' knowledge, attitudes, and practices regarding deleterious oral habits leading to malocclusion in children.

In orthodontics, malocclusion is a misalignment or incorrect relation between the teeth of the upper and lower dental arches when they approach each other as the jaws close. Edward Angle (1855-1930), the "father of modern orthodontics," popularized the term. The word derives from mal, which means "incorrect," and occlusion, which means "how opposing teeth meet."

Angle class I molar classification, also known as neutroclusion, is determined by the mesiobuccal cusp of the maxillary first molar occluding with the buccal groove of the mandibular first molar. A class II molar classification is a resolution, and it is determined by the mesiobuccal cusp of the maxillary first molar, occluding mesial to the buccal groove of the mandibular first molar. Lastly, a class III molar classification, also known as dissolution, can be determined by the mesiobuccal cusp of the maxillary first molar, occluding distal to the buccal groove of the mandibular first molar.

Several skeletal causes of malocclusion may require surgery, including jaw discrepancies, facial asymmetry, cleft lip and palate, and craniofacial abnormalities. Additionally, although much less common, injury to the face or jaw can cause malocclusion. It is important to note that the surgical treatment of malocclusion is typically only recommended in severe cases. Orthodontic treatment using braces is the first option in mild to moderate malocclusion cases. A thorough orthodontist or oral and maxillofacial surgeon evaluation is needed to determine the most appropriate treatment approach.

Oral habits leading to malocclusion are usually seen in the mixed dentition period, which is 8-12 years of age. At such an age, children are placed in schools for education. They spend nearly 6-7 hours per day on the school premises. Teachers are next to parents who can observe children's oral habits at school. If such school teachers know that oral habits can deteriorate their oral health, they can help prevent such damage. Hence, the study chose the school children group to know their levels of knowledge, attitude, and awareness toward oral habits.

Few studies assess the knowledge and attitude of school teachers regarding dental caries and malocclusion, and there is no literature on primary school teachers and oral habits leading to malocclusion. This is the first study to highlight the importance of primary school teachers in preventing oral habits leading to malocclusion.

## Materials and methods

Methodology

Study Setting and Design

One hundred fifty primary school teachers in the urban area of Nellore were randomly selected. The inclusion criteria were primary school teachers willing to participate in the study. Standard validated questionnaires from previously published studies were chosen. Eighteen questions were selected as per the study's objectives. There are six questions per category: knowledge, attitude, and practice assessment questions. The questionnaire was distributed, and the filled questionnaire was collected on the same day. One hundred fifty questionnaire forms were distributed, and the response rate was 100%.

The raw data obtained after assessing the questionnaire as yes or no will be entered into the SPSS (IBM Corp., Armonk, NY) statistical software data sheet, which transforms the data into percentages for ease of qualitative assessment. The data thus obtained in terms of percentages were analyzed statistically.

## Results

Demographic details

The demographic details of the respondents included were all women (100%) working in private schools in Nellore city, and they were selected based on a convenient sampling method.

A total of 150 teachers were selected from primary schools and included in the study. A knowledge-, attitude-, and awareness-based questionnaire was distributed to all teachers, and the response rate was 100% (n=150).

In the selected primary schools, 205 teachers were identified, out of which 150 volunteered to participate, with a response rate of 73.1%. Of the total population, 100% were women, with a mean age of 25.18±5.67 and a mean teaching experience (in years) of 3.40±2.36. Fifty percent of teachers have an essential educational degree (Bachelor of Education {BEd}); primary school teachers can identify the oral habits and malalignment of teeth, which is a crucial point to consider as it can help plan the prevention methods. Teachers need to get some training to become experts in identifying the psychological behavior that thrashes the child to adapt.

The results of the knowledge domain obtained for the question are presented in Table 1.

**Table 1:** Knowledge questions and their responses

Question	Yes	No
Do you know about oral habits?	30 (20%)	120 (80%)
Do you know the ill effects of long-standing oral habits?	54 (36%)	96 (64%)
Do you know about malocclusion?	62 (41.3%)	88 (58.7%)
Do you know oral habits cause malocclusion?	63 (42%)	87 (58%)
Do you know these are oral habits?	67 (44.7%)	83 (55.3%)
Do you know at what age permanent teeth start erupting?	75 (50%)	75 (50%)

Only 30 participants (20%) said they knew about oral habits, but 96 (64%) participants did not know about the ill effects of long-standing oral habits. Only 62 (41.3%) know about malocclusion, and 87 (58%) know that oral habits cause malocclusion. Only 67 (44.7%) know all oral habits, and among the 150 participants, half of them, that is, 75 of them, know at what age permanent teeth start erupting. In their study, Rai et al. examined the knowledge and attitudes of primary school teachers [7]. They discovered that 69.94% of their study populations were unaware of oral habits, almost the same as this study.

The attitude domain results are presented in Table 2.

**Table 2:** Attitude questions and their responses

Questions	Yes	No
Did you observe any deleterious oral habits in children?	104 (69.3%)	46 (30.7%)
Did you notice any abnormal alignment in children?	99 (66%)	51 (34%)
Do you like to know about the disbenefits caused by pernicious oral habits?	110 (73.3%)	40 (26.7)
Do you know why children develop oral habits?	65 (43.3%)	85 (56.7%)
Did you notice any difference in the psychological behavior of the children noticing their friends having oral habits?	57 (38%)	93 (62%)
Did you notice observational learning in other children by observing the other children?	87 (58%)	63 (42%)

Among 150 teachers, 104 (69.3%) observed the harmful effects of oral habits in children, with 99 (66%) noticing the abnormal alignment of teeth in the dentition of children. One hundred ten (73.3%) knew the disbenefits caused by pernicious oral habits. Sixty-five (43.3%) said they consider why children develop such habits. Only 57 (38%) noticed that children have some psychological and behavioral differences in those who perform the habits. Eighty-seven (58%) of the teachers noticed that these habits were part of observational learning and that children already had oral habits.

The practice domain results are presented in Table 3.

**Table 3:** Practice questions and their responses

Questions	Yes	No
Did you stop the children from doing these habits?	110 (73.3%)	40 (26.7%)
Do you educate children about the adverse effects caused by deleterious oral habits?	59 (39.3%)	91 (60.7%)
Do you explain to the parents about the oral habits of their children?	74 (49.3%)	76 (50.7%)
Do you refer parents to consult a dentist about the habits?	73 (48.7%)	77 (51.3%)
Did you notice any difference in children after educating them about the oral habits?	52 (34.7%)	98 (65.3%)
Do you practice toothbrushing in children?	57 (38%)	93 (62%)

Out of 150 teachers, 110 (73.3%) stopped the children from making these habits. Of the teachers who stopped the children from making these habits, only 59 (39.3%) educated the children about the adverse effects caused by harmful oral habits. Fifty-two (34.7%) noticed a difference in children after educating them; 74 (49.3%) explained to the parents about the oral habits of their children, and 73 (48.7%) knew that they had to refer the parents to consult a dentist about the habits. This study also considered the practices of performing oral hygiene at school, and only 57 (38%) involved themselves in making the children perform brushing as part of the oral hygiene practices.

The results of this study showed that nearly 80% of primary school teachers lack knowledge regarding oral habits. This proves that there is a need to train primary school teachers regarding oral health and its maintenance.

The study's results suggested that dental organizations frame the training protocol and design training methods such as video conferences, workshops, and educational skits in schools.

Only 40% of the school teachers could educate the children, while the others probably need more knowledge about the deleterious effects of oral habits. Fifty percent of teachers tried to explain to the children's parents, taking it as their responsibility to inform them about oral habits. They have made this move because they think that the parents should be informed so that they will take enough care of the children.

Among the 150 participants, only 49% referred these children to the dentist as they did not have enough knowledge about consulting a dentist, and this was proved in the study results. The results indicate the need to create awareness among school teachers regarding the role of dentists.

## Discussion

Oral habits such as thumb-sucking, nail biting, and tongue thrusting will hurt dentition leading to malocclusion in children. These habits tend to develop during primary school and show their effects through permanent dentition [7]. Children of this age usually develop these habits because of insecurities. These study results reveal the association between each domain and every question used to determine the knowledge, attitude, and practices of primary school teachers regarding children's oral habits, which may have harmful effects on the development of dentition [8].

The results of this study indicate that most, that is, 80% of primary school teachers, lack knowledge of oral habits. About 36%-45% know about malocclusion and the ill effects of malocclusion and that malocclusion is the effect of long-standing oral habits. Only 50% know at what age permanent teeth start erupting. If one knows about the eruption age, it will become easy to explain the association of these habits to cause disturbances in the next establishing dentition. By the qualitative assessment of the results through this domain, school programs need to be planned to educate and demonstrate what oral habits are and the effects of oral habits on developing dentition [9].

The results of the attitude domain are presented in Table 2. About 70% of the teachers have observed deleterious oral habits in children, and 66% observed abnormal teeth alignment in children. In comparison, only 38% of them noticed that it psychologically impacts the other children's development of oral habits [10]. While 58% thought it was just the impact of observational learning, that is, by observing other children having oral habits, most of them learn those habits. The attitude-revealing questions made it clear that very few school teachers know that oral habits result from psychological impact on school children. Public health sectors need to visit primary schools to make the same clear and train them to address students' insecurities well before they can impact their psychology and develop oral habits [11].

The results of the practice-based domain are presented in Table 3. The results of the practice-based questions show that about 74% of school teachers stop children from engaging in these habits. This shows good intent and concern for their students. Only 40% educate the children as they probably think that they cannot understand the reason for themselves to come out of it and, if they also educate, it is of no use and a waste of time. Fifty percent explain to the parents that they think it is their responsibility to tell them and that they should ultimately bear the responsibility of their children to see that they are normal without any physical disturbances [12]. Among the 150 participants, only 49% referred these children to the dentist as they did not know about referring them to the dentist, and this was also very obvious and proven through this study [13,14]. Following this would benefit the children and help them to cease these habits, which can benefit as the dentist would address children's insecurities and break the habit with a habit-breaking appliance. Only 34.7% noticed a difference in the children after educating them about oral habits. However, suppose the second and fifth practice questions are observed closely. In that case, there is an association that only 40% of the children were educated and 34.7% stopped the habit after education [15,16]. As oral health is overall health, we must stress the importance of establishing oral habits. If they are not addressed, their insecurities will lead to malocclusion and inefficient chewing.

Limitations of the study

The same research should be continued and implemented with a larger sample in different schools, such as rural and urban schools and private and government schools, with other boards such as the state syllabus/Central Board of Secondary Education (CBSE)/Indian Certificate of Secondary Education (ICSE), and the difference between the studied groups should be analyzed.

The operator was not blinded to the examination and collection of data, and the same operator led the survey process.

The study has helped evaluate the primary school teachers' knowledge, attitude, and practice of identifying and halting the habit in children, which will help develop dental health education programs well suited to their knowledge level. Another disadvantage of our study is that we did not collect the teachers' educational qualifications; such criteria would have a significant impact on the amount of information the teachers had. There was no blinding because only one examiner collected the data and conducted the survey.

Implications for the future

This study has assisted us in evaluating the primary school teachers' knowledge, attitude, and practice of identifying and halting the habit in children, which will help develop dental health education programs well suited to their knowledge level. If the teachers passed on the knowledge, this would help raise parental understanding of what to watch for. After the study with the groups mentioned above, the individual modules can be developed according to the study results. All these strategically focus on the research ideas and objectives, which are to completely stop children from using oral habits as it has already been proven that they have deleterious effects on the development of dentition.

## Conclusions

Considering the study's limitations, it can be concluded that training modules need to be developed to impact the primary school teachers' cognitive, affective, and psychomotor domains about the harmful effects of oral habits to prevent malocclusion. The results of the study show that school teachers lack knowledge about oral habits. We need to focus on educating more about what oral habits are, the ill effects of long-standing oral habits on malocclusion, and how oral habits lead to malocclusion. We need to emphasize why primary school teachers need to learn about all these, which will impact the development of the permanent dentition.
